# Severe Corrosion of Carbon Steel in Oil Field Produced Water Can Be Linked to Methanogenic Archaea Containing a Special Type of [NiFe] Hydrogenase

**DOI:** 10.1128/AEM.01819-20

**Published:** 2021-01-15

**Authors:** Sven Lahme, Jaspreet Mand, John Longwell, Ramsey Smith, Dennis Enning

**Affiliations:** a ExxonMobil Upstream Research Company, Spring, Texas, USA; Wageningen University

**Keywords:** microbially influenced corrosion, biofilms, hydrogenase, methanogenesis

## Abstract

Methanogenic archaea have long been implicated in microbially influenced corrosion (MIC) of oil and gas infrastructure, yet a first understanding of the underlying molecular mechanisms has only recently emerged. We surveyed pipeline-associated microbiomes from geographically distinct oil field facilities and found methanogens to account for 0.2 to 9.3% of the 16S rRNA gene sequencing reads. Neither the type nor the abundance of the detected methanogens was correlated with the perceived severity of MIC in these pipelines. Using fluids from one pipeline, MIC was reproduced in the laboratory, both under stagnant conditions and in customized corrosion reactors simulating pipeline flow. High corrosion rates (up to 2.43 mm Fe^0^ · yr^−1^) with macroscopic, localized corrosion features were attributed to lithotrophic, mesophilic microbial activity. Other laboratory tests with the same waters yielded negligible corrosion rates (<0.08 mm Fe^0^ · yr^−1^). Recently, a novel [NiFe] hydrogenase from Methanococcus maripaludis strain OS7 was demonstrated to accelerate corrosion. We developed a specific quantitative PCR (qPCR) assay and detected the gene encoding the large subunit of this hydrogenase (labeled *micH*) in corrosive (>0.15 mm Fe^0^ · yr^−1^) biofilms. The *micH* gene, on the other hand, was absent in noncorrosive biofilms, despite an abundance of methanogens. Reconstruction of a nearly complete Methanococcus maripaludis genome from a highly corrosive mixed biofilm revealed *micH* and associated genes in nearly identical genetic configuration to that in strain OS7, thereby supporting our hypothesis that the encoded molecular mechanism contributed to corrosion. Lastly, the proposed MIC biomarker was detected in multiple oil fields, indicating a geographically widespread involvement of this [NiFe] hydrogenase in MIC.

**IMPORTANCE** Microorganisms can deteriorate built environments, which is particularly problematic in the case of pipelines transporting hydrocarbons to industrial end users. MIC is notoriously difficult to detect and monitor and, as a consequence, is a particularly difficult corrosion mechanism to manage. Despite the advent of molecular tools and improved microbial monitoring strategies for oil and gas operations, specific underlying MIC mechanisms in pipelines remain largely enigmatic. Emerging mechanistic understanding of methanogenic MIC derived from pure culture work allowed us to develop a qPCR assay that distinguishes technically problematic from benign methanogens in a West African oil field. Detection of the same gene in geographically diverse samples from North America hints at the widespread applicability of this assay. The research presented here offers a step toward a mechanistic understanding of biocorrosion in oil fields and introduces a binary marker for (methanogenic) MIC that can find application in corrosion management programs in industrial settings.

## Abstract (precis)

Microorganisms can deteriorate built environments, which is particularly problematic in the case of pipelines transporting hydrocarbons to industrial end users. MIC is notoriously difficult to detect and monitor and, as a consequence, is a particularly difficult corrosion mechanism to manage.

## INTRODUCTION

Microbially influenced corrosion (MIC) is problematic for oil field operations when it leads to extensive damage requiring the repair or replacement of infrastructure such as carbon steel pipelines. While some metal loss is expected and accounted for during the design of the infrastructure (e.g., 0.1 mm · yr^−1^ for a pipeline designed with a 3-mm corrosion allowance for a 30-year design life), higher rates of corrosion, unless detected and mitigated in a timely manner, can necessitate costly repairs or replacements long before the design service life has been actualized.

The most common oil field corrosion mechanisms are linked to CO_2_ and/or hydrogen sulfide (H_2_S), which are present in oil-bearing formations and readily dissolve into the water that is associated with production fluids (termed produced water). The deleterious effects of these acid gases are managed through injection of corrosion inhibitors (CIs), typically film-forming surfactants that act as a diffusion barrier between steel and the aqueous corrosive environment (1, 2). Acid gas corrosion can be modeled *in silico* (3) or simulated in laboratory reactors (4) to understand the rates of metal loss expected under specific field conditions (i.e., flow, temperature, brine chemistry, and partial pressure of acid gases). Furthermore, laboratory testing provides a robust approach to ensure CI effectiveness (4, 5). Microbial corrosion, on the other hand, cannot be adequately modeled, and laboratory qualification of oil field biocides, applied to control MIC, typically falls short at predicting product effectiveness in the field. As a consequence, field-based detection and monitoring is particularly important for effective management of this corrosion mechanism.

In environments devoid of oxygen, MIC has been linked to a variety of anaerobic microorganisms (see reference 6 and references therein); however, most attention has historically been given to the sulfate-reducing bacteria (SRB) due to their well-documented ability to cause severe corrosion in laboratory studies and their prevalence in production systems (see reference 7 and references therein). SRB impact metal integrity by producing hydrogen sulfide (H_2_S) as a corrosive metabolic end product of sulfate reduction with (usually) organic electron donors. A small number of lithotrophic SRB isolates have further been shown to severely accelerate corrosion of steel in laboratory tests by utilizing cathodic electrons for their metabolism (8–10). It was proposed that outer membrane *c*-type cytochromes enable the direct uptake of electrons from steel in one such isolate, Desulfovibrio ferrophilus strain IS5 (11). In addition to SRB, methanogenic archaea are also increasingly viewed as prime culprits in the corrosion of steel (12–19). Some methanogens have been shown to utilize metallic iron (Fe^0^) in carbon steel as the sole electron donor for methanogenesis (13, 14, 19, 20). Electrochemical studies suggested a mediator-free direct electron uptake mechanism for one of these Fe^0^-utilizing isolates, Methanobacterium-like strain IM1 (21). The potential technical relevance of this microorganism has been demonstrated in laboratory studies in which strain IM1 was grown in flow-through systems (22).

A deeper understanding of the underlying biochemical basis of Fe^0^ utilization by methanogenic archaea has recently been provided. By working with filtered spent culture medium, as well as hydrogenase-negative mutants of Methanococcus maripaludis strain MM901, Deutzmann and colleagues demonstrated that free, steel-associated hydrogenases from this strain catalyzed the production of hydrogen (H_2_) from Fe^0^ granules (23). Whether these hydrogenases originated from partial lysis of the laboratory culture (e.g., in the stationary phase) or were actively excreted is currently unknown. Likewise, it is unclear whether this mechanism can produce technically relevant MIC in laboratory or oil field settings. Tsurumaru and colleagues performed comparative genomics on multiple strains of Methanococcus maripaludis and identified a 12-kb genomic region (termed the “MIC island”) that was unique to pure cultures of strain OS7, the only strain of M. maripaludis that accelerated Fe^0^ oxidation (corrosion) in their tests (24). This MIC island encoded a novel type of [NiFe] hydrogenase, along with an extracellular transport system. It was postulated that the secreted [NiFe] hydrogenase would catalyze the reduction of H^+^ to molecular hydrogen on iron surfaces, thereby accelerating the oxidation of the provided iron granules.

These recent insights into the molecular mechanisms of MIC by methanogenic archaea prompted us to investigate the role of methanogens in the corrosion of oil and gas infrastructure. Since MIC is a biofilm-associated phenomenon (25), we began by surveying the archaeal microbiomes of steel-associated solids from pipelines in North America, East Asia, and West Africa. We then performed a comprehensive study of MIC using produced waters from one of these locations in controlled laboratory reactors that allowed the growth of methanogenic archaea. The extent of corrosion, as well as the associated microbial communities, was investigated under various enrichment conditions with natural oil field waters, as well as with synthetic brines to specifically enrich lithotrophic microorganisms. Building on these laboratory tests, we gained insights into the molecular mechanisms of MIC through shotgun DNA sequencing and metagenomic analysis of a highly corrosive, lithotrophic biofilm grown under simulated pipeline conditions. Lastly, we developed a novel quantitative PCR (qPCR) assay for the detection of the above-mentioned [NiFe] hydrogenase and demonstrated the applicability of this proposed MIC biomarker assay in industrial settings.

## RESULTS

### Methanogenic archaeal communities on carbon steel pipeline walls.

**FIG 1:**
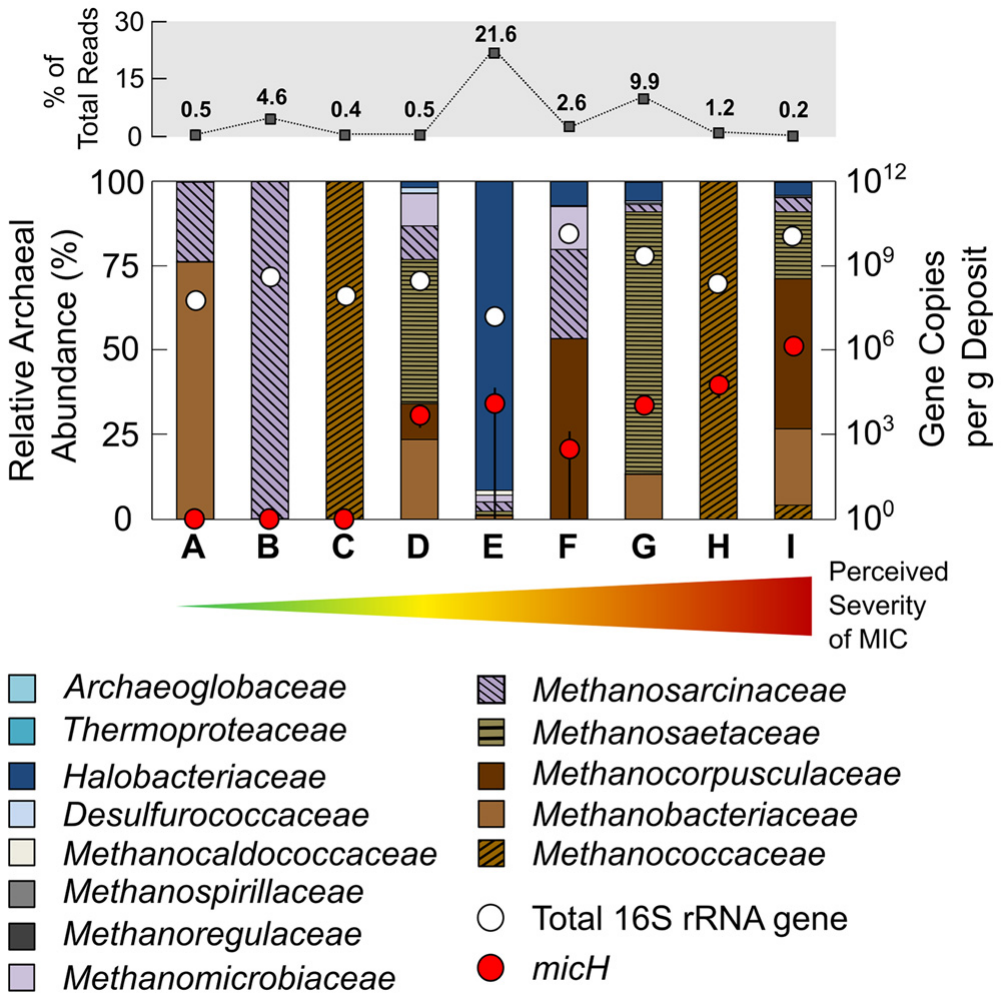
Archaeal community composition in solid samples (pig debris) collected from carbon steel pipelines. Archaeal sequences accounted for between 0.2 and 21.6% of all reads from 16S rRNA gene sequencing of DNA extracted from pipeline solids (top row). Total 16S rRNA gene numbers (archaeal plus bacterial) and gene copy numbers of the proposed methanogenic MIC biomarker *micH* are also plotted for each sample. Pipelines A to I are ordered according to their perceived severity of MIC (increasing from left to right, as indicated by arrow). The perceived (qualitative) severity of MIC is based on in-line inspection [ILI] data, field and laboratory studies, and operator experience. (A) Offshore multiphase pipeline (East Asia). (B) Offshore crude transmission pipeline (U.S. Gulf of Mexico). (C) Offshore multiphase pipeline (U.S. West Coast). (D) Onshore crude transmission pipeline (U.S. Gulf Cast). (E) Onshore crude transmission pipeline (U.S. West Coast). (F) Onshore produced water pipeline (U.S. Midwest). (G) Offshore multiphase pipeline (Nigeria). (H) Offshore multiphase pipeline (U.S. West Coast). (I) Offshore multiphase pipeline (Nigeria). The error bars depict the standard deviation of technical replicates (*n* = 3) of the *micH* quantitative PCR (qPCR) analysis.

Maintenance of oil field pipelines involves the periodic use of devices with blades or brushes (termed “pig” [pipeline intervention gadget]) to mechanically clean out rust, wax, scale, and biofilm (collectively termed “pig debris”). This practice offers the opportunity to access steel-associated microorganisms from pressurized hydrocarbon pipelines. We obtained pig debris from nine different pipelines in seven geographically distinct oil field operations in North America, East Asia, and West Africa to survey the prevalence of methanogenic archaea (Fig. 1). Estimated 16S rRNA gene abundances of 1.6 × 10^7^ to 1.3 × 10^10^ gene copies per g of pig debris suggested moderately dense microbial colonization of pipe wall-associated solids. Archaeal sequences accounted for 0.2 to 21.6% of total reads, and most of the detected archaea were classified as members of methanogenic archaeal families (0.2 to 9.3% of total reads). Members of the Methanococcaceae, Methanobacteriaceae, and Methanosarcinaceae have been implicated in MIC through laboratory investigations (13, 17, 19, 24). However, there were no apparent correlations between the types or abundances of methanogens and the perceived severity of MIC in the surveyed pipelines (Fig. 1). For example, pipelines A to C had no indications of active internal corrosion (MIC or otherwise) in recent in-line inspections (ILI) (data not shown), despite the presence of microorganisms affiliated with the above-mentioned putatively corrosive archaeal families.

### Assessment of MIC under mesophilic and thermophilic conditions.

**FIG 2:**
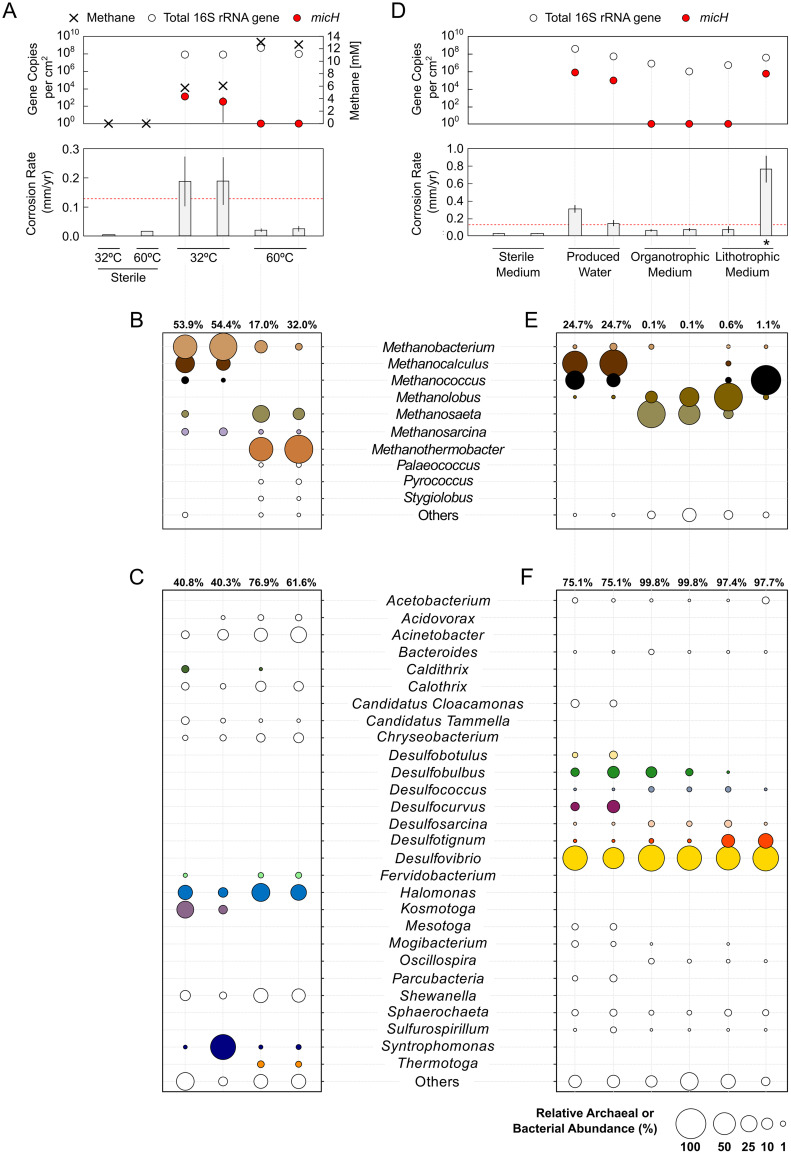
Growth of steel-attached oil field biofilms in bottle tests. (A to C) Tests with sulfate-free anoxic produced water obtained from the West African oil field and incubated under mesophilic (32°C) and thermophilic (60°C) conditions. (D to F) Tests with sulfate-amended produced water, as well as synthetic produced water medium (plus sulfate) in the presence or absence of propionate and acetate (organotrophic and lithotrophic conditions, respectively). (A and D) Averaged weight loss corrosion rates (CR), total 16S rRNA gene (archaeal and bacterial) quantification, and gene copy numbers of the proposed archaeal MIC biomarker *micH*. A threshold denoting technically relevant, high corrosion rates (≥0.13 mm Fe^0^ · yr^−1^) is indicated by the dashed red line. Methane formation is depicted as the final concentration in headspace after 3 months (A). Archaeal (B and E) and bacterial (C and F) community composition was assessed by 16S rRNA gene sequencing of DNA extracted from biofilms grown on carbon steel coupons. Numbers above the abundance plots indicate the percentage of total sequencing reads. Archaeal sequences accounted for 0.1 to 54.4% and bacterial sequences for 40.3 to 99.8% of the total sequencing reads. Genera with abundances of <1% in either the bacterial or archaeal fraction of all biofilm samples were merged under “Others.” The enrichment culture that was used for a subsequent lithotrophic corrosion kettle test is indicted by an asterisk. The error bars depict the standard deviation of technical replicates (*n* = 3) for weight loss corrosion rates and *micH* qPCR analysis.

The detection of methanogenic archaea in all surveyed pipelines necessitated a more nuanced understanding of their potential role in corrosion, a question that was approached through laboratory cultivation. We hence obtained anoxic production fluids from a West African oil field with a history of MIC (Fig. 1, pipelines G and I). Carbon steel infrastructure typically spans broad temperature gradients within the same oil field due to hot production fluids from reservoirs (often ≫60°C) cooling as they pass through networks of pipelines, pressure vessels, and piping. Fluids from subsea pipeline I had an average temperature of 32°C at the time of sampling. In the laboratory, the sulfate-free (<20 μM) produced water was transferred into glass bottles with specially designed carbon steel coupon holders with rubber stoppers and an anoxic headspace of N_2_-CO_2_. Bottles were incubated at 32°C and 60°C on rotary shakers (75 rpm) for 13 weeks under conditions that are most representative of process dead legs or idled pipelines (i.e., stagnant to very low flow). Indeed, methanogenic biofilms developed on carbon steel surfaces exposed to the sulfate-free produced water, as evidenced by accumulation of CH_4_ in the test bottles (Fig. 2A) and detection of high numbers of steel-attached methanogenic archaea through qPCR and 16S rRNA gene sequencing (Fig. 2A and B). Archaea accounted for 17.0 to 54.4% of the steel-attached biofilms. Archaeal communities grown at 32°C showed high abundances of hydrogenotrophic (i.e., lithotrophic) Methanobacterium spp. and Methanocalculus spp., with minor fractions of Methanococcus spp. Incubation at higher temperatures (60°C) led to formation of biofilms with prominent fractions of hydrogenotrophic *Methanothermobacter* spp. and, at lower prevalence, the acetoclastic Methanosaeta spp. (Fig. 2B). Mesophilic bacterial communities (32°C) contained high fractions of Syntrophomonas spp., Kosmotoga spp., and Halomonas spp. that might have grown through fermentation of residual organic compounds in the produced water, thereby producing acetate, CO_2_, and H_2_ (26–28). The majority of the bacterial biofilm community in bottles incubated at 60°C consisted of mesophilic microorganisms (Fig. 2C). However, some thermophilic bacteria, such as Thermotoga, Fervidobacterium, and Caldithrix were detected and could have grown via a fermentative metabolism (29–31). Consistent with the lack of sulfate in these natural oil field waters, we only detected minor fractions of SRB in steel-attached biofilms (cumulatively, <0.6% per biofilm). The different incubation temperatures resulted in markedly different metal damage (Fig. 2A). Microbial activity had a negligible impact on carbon steel at 60°C, with an increase in corrosion rates of approximately 40% compared to sterile controls, but a low average corrosion rate of 0.02 ± 0.01 mm Fe^0^ · yr^−1^. Mesophilic microorganisms, on the other hand, accelerated corrosion by a factor of 12 and led to localized damage with features as deep as 399 μm developing over 3 months (Fig. 2A and Fig. S1 in the supplemental material). The National Association of Corrosion Engineers (NACE) categorizes corrosion rates of carbon steel coupons of >0.13 mm Fe^0^ · yr^−1^ as “high” and those of >0.25 mm Fe^0^ · yr^−1^ as “severe” (32). Interestingly, despite the profoundly different impacts on corrosion, biofilms grown at 32°C and 60°C contained similar numbers of active methanogens (Fig. 2A). This observation implied that intrinsic properties of the mesophilic biofilm community, rather than the overall number of cells or methanogenic activity, were linked to the observed acceleration of corrosion kinetics.

### Identification of potential methanogenic corrosion mechanisms.

Tsurumaru and colleagues recently linked the accelerated oxidation of Fe^0^ (i.e., corrosion) in cultures of *M. maripaludis* strain OS7 to a novel [NiFe] hydrogenase (24). A protein sequence comparison of the large subunit of this hydrogenase against the National Center for Biotechnology Information (NCBI) protein sequence database revealed that three sequenced members of the Methanobacteriales contain similar proteins (90.7 to 93.5% protein sequence identity), while all other deposited sequences showed ≤40% protein sequence identity (see Table S1 in the supplemental material). The high degree of protein sequence resemblance suggested functional similarity within this protein cluster and prompted us to develop a specific qPCR assay. We developed a degenerated primer pair and TaqMan probe (Table 1) to detect and quantify the gene of the large subunit of the [NiFe] hydrogenase (referred to here as *micH*) in strain OS7 and the three other methanogenic strains. This assay allows for probing of mixed microbial biofilms for their ability to carry out a proven microbial corrosion mechanism, i.e., the enzymatic catalysis of Fe^0^ oxidation via an extracellular hydrogenase. Intriguingly, we could detect 5.4 × 10^2^ and 1.9 × 10^3^ *micH* gene copies per cm^2^ in corrosive biofilms at 32°C, whereas the *micH* gene was undetectable in noncorrosive biofilms grown at 60°C (Fig. 2A). This raised the possibility that mesophilic, lithotrophic methanogens, likely of the genera *Methanococcus* or *Methanobacterium*, had caused the observed high rates of corrosion.

**TABLE 1:** Sequences of degenerate primer pair and probe used in qPCR assays to detect the proposed methanogenic MIC biomarker *micH*

Primer or probe^*a*^	Sequence (5′→3′)^*b*^
micH-fwd	AA(Y)CTTCTAACACCAA(M)TGA(Y)GGAAC
micH-rev	TCAAATCCTCTAAATTCTGTGACACA
micH-probe	TACCAACAGATAATGCTGCA

a Proposed product length, 95 bp.

b M = A or C; Y = C or T.

### Enrichment of organotrophic and lithotrophic oil field communities.

We sought to reproduce our observations and collected a new batch of production fluids from the same West African oil field. Traces of H_2_S (approximately 100 ppm) measured in the gas phase of the sample collection drums upon arrival in our laboratories were indicative of sulfate reduction, so we amended the next tranche of corrosion bottle tests with sulfate to allow the growth of SRB. Again, highly corrosive mesophilic biofilms developed in these produced waters, and putatively corrosive *Methanococcus* spp., as well as *Methanocalculus* spp., were detected in large fractions (Fig. 2D and E). Furthermore, these biofilms contained *micH* at even higher numbers than previous cultures, with 1.0 × 10^5^ to 9.9 × 10^5^ gene copies per cm^2^ (Fig. 2D).

The use of actual produced waters for laboratory studies offers the opportunity to closely simulate oil field MIC in laboratory settings. However, it also complicates data interpretation, since the presence of complex organic matter can allow a multitude of fermentative and oxidative metabolisms. We therefore dissected experimental conditions by working with defined cultivation media which were modeled after the major ion composition of the original produced water (plus sulfate). Organotrophic and lithotrophic enrichment cultures containing steel coupons were set up with or without addition of organic acids (12.1 mM acetate and 1.6 mM propionate), respectively. This reduced the number of potential biological transformations and, in the case of tests without the organic acids, limited growth to those microorganisms capable of utilizing metallic iron (Fe^0^) as an electron donor. Transfer of produced water as inoculum (0.5% vol/vol) into synthetic medium reduced microbial corrosion rates to moderate rates (<0.08 mm Fe^0^ · yr^−1^) in all but one of the bottle tests (Fig. 2D). Remarkably, this lithotrophic culture caused corrosion at severe rates of 0.76 ± 0.15 mm Fe^0^ · yr^−1^, with up to 370-μm deep corrosion features developing on carbon steel coupons over 3 months (Fig. 2D and Fig. S2 in the supplemental material). All biofilms in the synthetic produced water medium were numerically dominated by sulfate-reducing bacteria, in particular by Desulfovibrio spp., Desulfobulbus spp., and Desulfotignum spp. (Fig. 2F). The distinguishing features of the severely corrosive lithotrophic biofilm, however, appeared to be the enrichment of a subpopulation of Methanococcus spp. and the detection of the special [NiFe] hydrogenase *micH* (Fig. 2D and E).

### Corrosion under simulated pipeline condition.

The preceding bottle tests demonstrated the potential for severe MIC in stagnant water bodies within the infrastructure of the investigated oil field. However, the pipelines that transport multiphasic fluids (e.g., from offshore production wells) usually experience high flow and, as a result, wall shear stresses, which greatly increase the rates of acid gas corrosion resulting from CO_2_ (33). Likewise, biofilm formation can be affected by the physical stresses imposed by flow (34).

**FIG 3:**
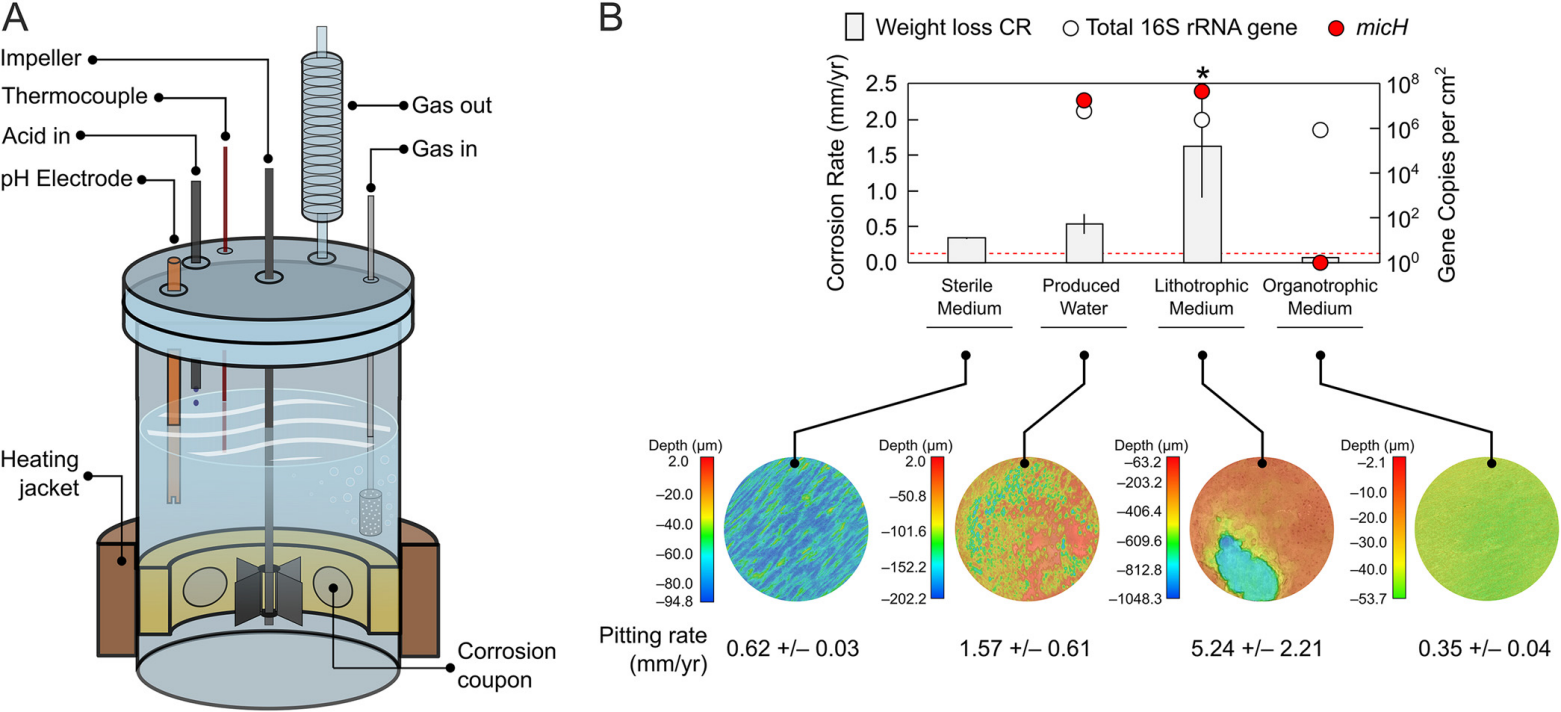
Growth of steel-attached oil field biofilms in corrosion kettle tests. (A) Schematic of customized corrosion kettles for the study of CO_2_ corrosion and MIC under pipeline-simulating conditions (flow and acid gas purging). (B) Averaged weight loss corrosion rates (CR) in anoxic kettle tests containing oil field microorganisms in West African produced water, as well as synthetic produced water medium with or without addition of the organic electron donors acetate and propionate (organotrophic and lithotrophic conditions, respectively). A sterile control test quantifying CO_2_ corrosion was also included. Total 16S rRNA gene (archaeal and bacterial) quantification and gene copy numbers of the proposed archaeal MIC biomarker *micH* in coupon biofilms are plotted for each test. A threshold denoting technically relevant, high corrosion rates (≥0.13 mm Fe^0^ · yr^−1^) is indicated by the dashed red line. False-color images show the topography of coupons at the end of the experiments after removal of corrosion products. Linearly extrapolated pitting corrosion rates calculated from average maximal feature depths are provided in the subpanel. The asterisk indicates the test selected for metagenomic analysis. The error bars depict the standard deviation of technical replicates (*n* = 3) for weight loss corrosion rates. Error bars from technical replication (*n* = 3) of *micH* qPCR analysis are smaller than the symbol size.

We therefore evaluated corrosion in customized test reactors (corrosion kettles) that allow for close simulation of pipeline conditions by controlling shear stress, pH, and acid gas composition (Fig. 3A). First, a test with sterile synthetic produced water was performed to establish the baseline corrosion from CO_2_. These tests revealed that abiotic conditions alone could cause severe corrosion of 0.34 ± 0.01 mm Fe^0^ · yr^−1^ (Fig. 3B and Fig. S3A in the supplemental material) under conditions that model pipelines G and I.

Still, inclusion of oil field microorganisms in these tests demonstrated the pronounced biotic component of pipeline corrosion under the studied conditions. A second test with actual produced water increased averaged corrosion rates beyond the CO_2_ baseline to 0.54 ± 0.14 mm Fe^0^ · yr^−1^ (Fig. 3B) and resulted in irregular and localized pitting corrosion at extrapolated rates of 1.57 ± 0.61 mm · yr^−1^ (Fig. 3B and Fig. S3A). Operation of a third corrosion kettle with lithotrophic produced water medium inoculated with a pre-enriched Fe^0^-utilizing culture (asterisk in Fig. 2D) resulted in even more aggressive metal damage. This culture catalyzed iron oxidation at rates of 1.62 ± 0.72 mm Fe^0^ · yr^−1^, leading to macroscopic localized corrosion features with an extrapolated growth rate of 5.24 ± 2.21 mm · yr^−1^ over 7 weeks (Fig. 3B). Microbially catalyzed metal oxidation at these rates, if left unmitigated, would profoundly challenge pipeline integrity.

High copy numbers of *micH* (>10^6^ copies per cm^2^) and high relative fractions of 28% and 42% of *Methanococcus* spp. were detected in biofilms grown in corrosion kettles operated with actual (organotrophic) and synthetic (lithotrophic) produced water, respectively (Fig. 3B and Fig. S3B). Other dominant microorganisms detected in corrosive biofilms in both reactors included *Desulfovibrio* spp. (Fig. S3B).

A fourth corrosion kettle was set up with synthetic produced water medium containing organic acids and was inoculated with an organotrophic subculture from the West African oil field. This test did not produce the previously observed high corrosion rates, despite the formation of similar biofilms, as measured by 16S rRNA gene abundance (Fig. 3B). Corrosion of coupons underneath these biofilms containing *Desulfovibrio*, *Desulfobulbus*, and Methanolobus species (Fig. S3B) was considerably lower than that in the sterile control, at 0.073 ± 0.001 mm Fe^0^ · yr^−1^ and resulted in rather uniform metal loss (Fig. 3B and Fig. S3A). This may be an indication that the biofilm limited CO_2_ corrosion by forming a passivating barrier, a phenomenon seen before in laboratory tests (see reference 6 and references therein). Notably, this “protective” biofilm did not contain any *Methanococcus* spp. or *Methanobacterium* spp. and tested negative for *micH* (Fig. 3B and Fig. S4 in the supplemental material).

### Genomes recovered from a severely corroded carbon steel coupon.

Shotgun metagenome analysis can be a useful tool to probe for blueprints of biochemical reactions in mixed microbial communities. We wanted to further investigate the molecular mechanisms of MIC seen in this study, and hence extracted and sequenced DNA of a biofilm that was (i) associated with severe corrosion, (ii) contained high fractions of potential culprit organisms such as *Methanococcus* spp. and *Desulfovibrio* spp., and (iii) was grown under pipeline-like conditions (see asterisk in Fig. 3B for the selected sample).

**FIG 4:**
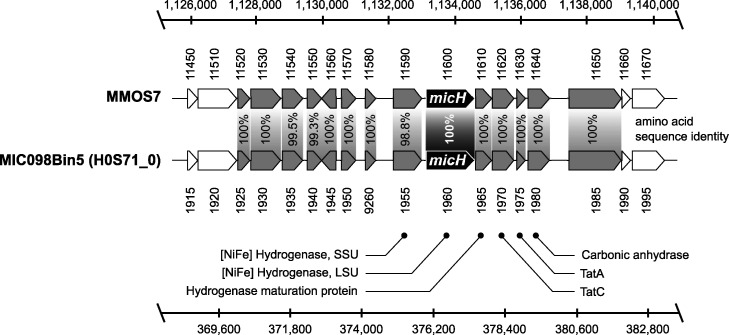
Comparison of the genetic arrangement of the MIC island in Methanococcus maripaludis strain OS7 (MMOS7) and the metagenome-assembled genome (MIC098Bin5) of an *M. maripaludis* strain grown on severely corroded steel surfaces. Scale bars indicate the nucleotide position in the respective genomes and the numbers below and above the genes refer to gene identifier numbers in the respective genomes, with prefixes of MMOS7 and H0S71_0, respectively.

We were able to obtain four nearly complete metagenome-assembled genomes (MAGs) from this sample (Table 2). Taxonomic classification by protein marker analysis indicated that one of the MAGs affiliated most closely with Methanococcus maripaludis. The *micH* gene in *M. maripaludis* strain OS7 is colocated on a 12-kb MIC island with genes hypothesized to be essential for the functioning and secretion of this hydrogenase (24). A sequence similarity search revealed that a *micH* homolog was indeed present in one of the assembled contigs of the *M. maripaludis* MAG. Furthermore, we discovered that the genetic arrangement of the MIC island in this MAG and strain OS7 was in fact identical (Fig. 4). Notably, the genetic island contained genes for both subunits of the putative [NiFe] hydrogenase, as well as the twin arginine translocation (Tat) system for secretion of the mature hydrogenase in strain OS7 (24). Protein sequence comparison further confirmed a high resemblance (98.8 to 100% amino acid sequence identity) between both clusters (Fig. 4).

**TABLE 2:** Metagenome-assembled genomes of methanogenic and sulfate-reducing microorganisms in severely corrosive biofilm

Taxonomic assignment^*a*^	Total sequence length (bp)	No. of contigs	*N*_50_ (bp)	*L*_50_	Completeness^*b*^ (%)	Contamination^*b*^ (%)
*Methanococcus maripaludis*	1,702,487	5	989,172	1	99.5	0.5
*Desulfovibrio alaskensis*	3,270,933	67	84,479	12	99.4	0.0
*Desulfovibrionaceae* bacterium	3,278,183	87	63,483	16	99.4	0.0
*Desulfovibrionaceae* bacterium	4,247,882	120	82,594	15	99.4	0.0

a Taxonomic assignment is based on GTDB-Tk classification.

b Completeness and contamination were estimated using CheckM.

Traditionally, SRB have been viewed as prime suspects of MIC (7). While all SRB can produce corrosive H_2_S under suitable organotrophic growth conditions, some specialized lithotrophic SRB strains have recently received much attention, as they can cause particularly severe corrosion through a more direct mechanism (8, 9, 13). It had been proposed that these organisms oxidize metal through direct electron uptake via outer membrane *c*-type cytochromes (OMCs) (13), with recent evidence supporting this hypothesis in D. ferrophilus strain IS5 (11). In this study, we obtained three nearly complete Desulfovibrionaceae genomes (Table 2). A reliable taxonomic classification was possible for one genome, which was assigned to a strain of Desulfovibrio alaskensis. However, despite the presence of three nearly complete *Desulfovibrionaceae* genomes, no evidence for an OMC system homologous to the one detected in strain IS5 was found in this metagenomic data set. This suggests that direct electron uptake via extracellular *c*-type cytochromes may not have contributed to MIC in our experiments.

The detection of *micH* in a metagenome-assembled genome from a highly corrosive biofilm further strengthened the applicability of this gene as a potential marker for MIC. We confirmed the presence of *micH* (up to 1.5 × 10^6^ gene copies per g) in pig debris from the West African oil field (pipelines G and I in Fig. 1). Intriguingly, we also detected 5.6 × 10^2^ to 5.6 × 10^4^ *micH* gene copies per g in four geographically separated pipelines in North America (Fig. 1).

## DISCUSSION

Methanogenic archaea are commonplace members of the oil field microbiome (16, 17, 35, 36). However, unlike SRB, which produce corrosive sulfides as a metabolic product, methanogenic archaea are not intrinsically corrosive (24, 37); they oxidize molecular hydrogen or a limited spectrum of small organic compounds and produce chemically inert CH_4_. We surveyed surface-associated deposits from nine pipelines in seven geographically distinct oil field operations and detected methanogenic archaea in all instances, despite vast differences in the perceived severity of MIC (Fig. 1). For example, methanogens affiliated with the *Methanobacteriaceae*, *Methanococcaceae*, and *Methanosarcinaceae* were detected in steel-associated solids from pipelines A to C by amplicon sequencing. Members of these archaeal families have been implicated in MIC through laboratory studies (13, 17, 19, 24), yet recent inspections of pipelines A to C did not reveal any indications of internal corrosion. Similarly, biofilms containing *Methanosaeta* spp., *Methanobacterium* spp., *Methanolobus* spp., and Methanothermobacter spp. caused only negligible corrosion (<0.08 mm Fe^0^ · yr^−1^) in laboratory reactors that simulated oil field conditions (Fig. 2 and 3 and Fig. S4). These data highlight that the mere presence of methanogenic biofilms on steel does not entail corrosion.

Corrosion of metallic iron in the absence of oxygen can be depicted by the following reaction equations (where *e*^−^ indicates electrons):

(1) $$Fe^{0}↔Fe^{2+}\;+\;2e^{-}$$

(2) $$2e^{-}\;+\;2H^{+}↔H_{2}$$

(3) $$Fe^{0}+\;2H^{+}\to Fe^{2+}\;+\;H_{2}$$

The anodic oxidation of iron (equation 1) is coupled to the cathodic reduction of a suitable oxidant, in anoxic environments usually H^+^ (equation 2), so that the net reaction involves formation of molecular H_2_ (equation 3). Reaction 2 is kinetically impeded, and as a result the corrosion of steel is slow in anoxic environment at neutral pH (9, 38, 39). However, corrosion in (slightly) acidic oil field waters can be pronounced in the presence of weak acids, as loosely bound protons from carbonic acid, organic acids, or sulfide dramatically increase the availability of H^+^ ions as oxidants at the steel surface, particularly in high flow scenarios (40).

Historic models of MIC had postulated that the consumption of cathodic molecular hydrogen by hydrogenotrophic microorganisms (including methanogens) would accelerate corrosion (41–43). However, this view has been disputed based on kinetic and thermodynamic considerations (8, 9, 44), as well as through copious experimental observations (8, 13, 14, 24, 37). However, while the majority of tested hydrogenotrophic methanogenic archaea do not influence steel oxidation to any significant extent, some strains have shown a pronounced impact on corrosion kinetics in laboratory experiments (13, 14, 24, 37). Recently, evidence has emerged that some pure cultures of methanogenic archaea accelerate corrosion when they produce extracellular hydrogenases that catalyze reaction 2 on steel surfaces (23, 24).

### Mesophilic methanogens in highly corrosive oil field biofilms contain a special MIC hydrogenase.

In this study, we reproduced oil field corrosion using fluids from an offshore production site in West Africa. Formation of mesophilic biofilms considerably increased corrosion rates over those in sterile controls, both in *de facto* stagnant bottle tests and in corrosion kettles simulating flow and field-like partial pressures of CO_2_ (Fig. 2 and 3). Technically highly relevant MIC rates (0.15 to 1.62 mm Fe^0^ · yr^−1^) and severe pitting were observed in several of these bioreactors. By using specially formulated synthetic produced water medium, we could further demonstrate that the observed Fe^0^ oxidation was in fact a lithotrophic process. Some hydrogenotrophic *Methanococcus* spp. and *Methanobacterium* spp. have been shown to effectively utilize Fe^0^ and were found in substantial numbers (>3.7 × 10^5^ 16S rRNA gene copies per cm^2^) in highly corrosive biofilms (Fig. 2 and 3; see also Fig. S4B in the supplemental material). However, both genera were also present in nearly all other (noncorrosive) biofilms, confirming that hydrogenotrophy itself does not accelerate corrosion kinetics.

Tsurumaru and colleagues recently discovered that only a few known strains of *M. maripaludis* and *Methanobacterium* spp. encode a unique hydrogenase that catalyzes the reduction of H^+^ to H_2_ on Fe^0^ granules and thereby considerably accelerates iron oxidation (24). The apparent high conservation among proteins from members of *Methanococcaceae* and *Methanobacteriaceae* allowed us to develop a specific qPCR assay targeting the gene of the large subunit, *micH*, of this hydrogenase. This assay did in fact distinguish biofilms causing technically relevant corrosion (>0.13 mm Fe^0^ · yr^−1^) from those that did not (<0.08 mm Fe^0^ · yr^−1^) in our experiments with actual and synthetic produced waters (Fig. 2 and 3 and Fig. S4B). *M. maripaludis* strain OS7 harbors this unique hydrogenase (*micH*) on a 12-kbp-long genetic island, along with several other genes assumed to be essential for a fully functional extracellular enzyme (24). We were able to retrieve a nearly complete genome of a Methanococcus maripaludis strain that carried *micH* in an identical genetic arrangement to that of strain OS7 (Fig. 4). This indicated that *micH* was associated with a corrosive Methanococcus maripaludis strain in our laboratory tests. Indeed, corrosive biofilms consistently contained both methanococcal 16S rRNA genes and the *micH* gene (Fig. S4B).

There was no linear correlation between the extent of corrosion and copy numbers of *micH* (Fig. S4). High corrosion rates were observed on coupons containing as few as 5.4 × 10^2^ gene copies per cm^2^ at the time of biofilm collection (Fig. 2A). It should be noted, though, that corrosion rates were averaged from metal loss over the entire experimental period (e.g., 3 months), while quantification and characterization of target genes in biofilms was only performed at test takedown and hence only represents endpoint conditions. Methanogens precipitate siderite (FeCO_3_), which can passivate steel surfaces (18, 45) and could have buried initially corrosive methanogens in some tests, possibly allowing for the degradation of “forensic evidence.” This could explain low copy numbers in some highly corrosive biofilms. More importantly, though, we do not envision gene copy numbers of *micH* to correlate with the final abundance of the actual enzyme that catalyzes iron oxidation. Still, *micH* in biofilms served as a binary genetic marker for technically relevant microbial corrosion in our tests with West African produced waters.

Methanogenic archaea constituted only a part, and in some instances a minor part, of the biofilm communities that developed under simulated oil field conditions. In addition to organic compounds in the produced waters and the organotrophic synthetic produced water medium (e.g., acetate and propionate), small quantities of cathodic hydrogen from abiotic corrosion, according to reaction 3, also served as a readily available electron donor, e.g., for sulfate-reducing bacteria. It is tempting to speculate that enzymatically catalyzed H_2_ formation from extracellular hydrogenases could have fed additional electron donors into the biofilm community. The potential of electroactive H_2_-forming microorganisms to act as “ecosystem engineers” by supporting the growth of nonelectroactive microorganisms in electrode biofilms has been previously proposed (46).

### The role of sulfate-reducing bacteria in corrosion.

The importance of sulfate-reducing bacteria in microbial corrosion has long been established (see reference 7 and references therein), and the underlying mechanisms as well as their regulation are still an active area of research (11, 47–49). While we cannot exclude the contribution of SRB to corrosion, we inferred that SRB likely played a minor role in our experiments. First, *Desulfovibrio* spp. were found at high abundances in all biofilms under sulfate-reducing conditions, but high or severe corrosion only occurred in the presence of *micH*-positive methanogens (Fig. 2D and 3B and Fig. S4). Second, high microbial corrosion rates were also observed in sulfate-free produced water (Fig. 2A). Third, only few lithotrophic SRB isolates have been demonstrated to cause corrosion to an extent similar to what was observed in this study (7, 8); the extracellular multiheme *c*-type cytochromes that have been linked to direct electron uptake in those corrosive strains (11) were absent in the metagenomic data set. The low to moderate corrosion rates in the absence of *micH*-positive *Methanococcus* spp. agree with corrosion rates documented for sulfidogenic cultures of D. alaskensis (≤0.1 mm Fe^0^ · yr^−1^; [22, 49, 50]). We cannot, however, exclude the possibility that hitherto unknown enzymatic mechanisms may have affected corrosion in our experiments.

### Proposed use of *micH* as a genetic marker for methanogenic MIC.

Microbial corrosion of oil field infrastructure is difficult to detect and monitor, in large part because the responsible microorganisms and underlying mechanisms remain enigmatic (51). For example, the mere presence of large numbers of methanogenic archaea in pipeline-associated debris or laboratory-grown biofilms proved to be a poor indicator of MIC in this study (Fig. 1 and Fig. S4). A deeper mechanistic understanding of MIC in methanogens and other environmental microorganisms offers a route to a more granular analysis and interpretation of microbiological monitoring data in oil fields and other industrial settings. Enzymatic acceleration of corrosion via hydrogenases is one such potentially relevant corrosion mechanism. This was recently evidenced through careful experimentation with pure cultures of methanogenic archaea (24) and was demonstrated in this study with mixed microbial communities in bioreactors simulating oil field corrosion. We designed an assay to quantify the gene encoding an extracellular hydrogenase (*micH*) in methanogens and demonstrated its ability to serve as a binary marker for MIC in experiments with oil field cultures grown under stagnant and pipeline flow-simulating conditions (Fig. S4B). The proposed MIC biomarker was detected in high concentrations (up to 1.5× 10^6^ gene copies per g) in pipeline-associated solids from a West African oil field with a history of MIC (pipeline I in Fig. 1). Interestingly, we also detected *micH* in similar solids from pipelines on the West Coast and Gulf Coast, as well as from the Midwest, of the United States (Fig. 1). The genetic island carrying this gene was first detected in *M. maripaludis* strain OS7, which originated from an underground oil storage tank in Japan, and it was further detected in the genome of Methanobacterium congolense Buetzberg by the same group (24). Strain Buetzberg was originally isolated from a biogas plant in Germany (52). The fact that we were able to reconstruct a nearly identical MIC island from a West African oil field strain of *M. maripaludis* (Fig. 4) suggests an extremely high level of conservation of the MIC island and the presence of these corrosive methanogens in industrial facilities across four continents. Future work may focus on developing a better understanding for the quantitative interpretation of *micH* in solid and liquid samples to enable early detection and mitigation of severe cases of microbially influenced corrosion in industrial settings.

## MATERIALS AND METHODS

### Collection of solid samples (pig debris) from pipelines.

Solids from nine carbon steel pipelines were collected between July 2014 and June 2017 to study the microbiomes within hydrocarbon-transporting infrastructure. Subsea pipelines G and I transport fluids from one oil field offshore Nigeria. This field produces oil and sweet natural gas (i.e., no H_2_S). Subsea pipelines C and H transport fluids from an offshore oil field on the U.S. West Coast. The other five pipelines each carry fluids from separate oil and gas fields on the U.S. West Coast (E), Midwest (F), and Gulf Coast (B and D), as well as from East Asia (A). Samples were collected following the (internal) mechanical cleaning of these pipelines with maintenance pigs that remove and push out surface-associated and settled debris. These solid samples (collected in the pig trap) were carefully transferred into sterile containers, immediately refrigerated with ice packs and frozen in common household freezers within 4 h of collection. Samples were then shipped frozen to Houston, TX, where they were stored at −80°C until further analysis.

### Bottle corrosion testing with original produced water.

Production fluids (oil with associated water and gas) were collected at a pipeline outlet (I) in a large oil field offshore Nigeria in December 2014. The fluids were filled into an internally polytetrafluoroethylene (PTFE)-lined steel drum (approximately 20 liters) all the way to the brim and capped gas-tight. In the laboratory, the content of the gas-tight drum was purged with N_2_-CO_2_ (79:21) to remove any biogenic H_2_S that had formed in the drum during transit (8 weeks). Thereafter, anoxic and sterile technique was used to transfer the gravity-separated produced water into 1-liter glass bottles (600 ml each; pH 6.6 to 6.7) with butyl rubber stoppers under an N_2_-CO_2_ (79:21) headspace. For sterile incubations, the capped produced water bottles were autoclaved (anaerobically) at 121°C for 20 min. Three sterilized carbon steel coupons (3 × 1.25 cm^2^) in a coupon holder made of polyether ether ketone (PEEK) were added to each incubation (see reference 53 for details). Bottles were placed on rotary shakers (75 rpm) and incubated at 32°C or 60°C for 13 weeks (see Table S2 in the supplemental material).

In March 2016, a second batch of production fluids was collected at a location further downstream from the same pipeline (I), shipped to the laboratory (transit time, 5 weeks), and processed as described above. Here, bottles with anoxic produced water (pH 6.6) were amended with 1 mM sodium sulfate (Na_2_SO_4_) and incubated at 32°C for 6 weeks (Table S2).

### Bottle corrosion testing with synthetic produced water medium.

To study microbial corrosion under more defined conditions, bottle tests with synthetic produced water medium were set up. The medium was modeled after the Nigerian produced water (collected in March 2016 and referred to here as PW-2016) and contained the following (all Sigma-Aldrich, reagent-grade chemicals in reverse osmosis deionized [RO-DI] water): NaCl (15.31 g/liter), MgCl_2_·6H_2_O (0.562 g/liter), CaCl_2_·2H_2_O (0.434 g/liter), KCl (0.121 g/liter), and NaBr (0.064 g/liter). Additionally, Na_2_SO_4_ (1.48 g/liter) was added to allow for microbial sulfidogenesis. The brine was aliquoted (600 ml) into 1-liter glass bottles, purged with N_2_-CO_2_ (79:21), sealed with butyl rubber stoppers, and autoclaved. After cooling, 24 ml/liter of a sterile 1 M NaHCO_3_ stock solution (equilibrated with pure CO_2_) was added. Subsequently, the brines were supplemented with thiamine hydrochloride (0.1 mg/ml in 25 mM phosphate buffer [pH 3.4]), a vitamin mixture (0.04 mg/ml 4-aminobenzoic acid, 0.01 mg/ml D(+)-biotin, 0.1 mg/ml nicotinic acid, 0.05 mg/ml Ca-D(+)-pantothenate, 0.15 mg/ml pyridoxin dihydrochloride, 0.04 mg/ml folic acid, and 0.015 mg/ml liponic acid in 10 mM phosphate buffer [pH 7.1]), riboflavin (0.025 mg/ml in 25 mM phosphate buffer [pH 3.4]), cyanocobalamin (0.05 mg/ml in deionized water), a trace element mixture (7.5 mM FeSO_4_, 0.8 mM CoCl_2_, 0.5 mM ZnSO_4_, 0.5 mM MnCl_2_, 0.15 mM Na_2_MoO_4_, 0.5 mM H_3_BO_3_, 0.1 mM NiCl_2_, and 0.01 mM CuCl_2_ in 100 mM HCl), selenite and tungstate (each 0.02 mM), potassium phosphate (1 M), and ammonium chloride (2 M) from sterile stock solutions (1 ml/liter each), prepared as described previously (54). For organotrophic test conditions, the medium was further supplemented with sodium acetate (NaCH_3_CO_2_; 0.996 g/liter) and sodium propionate (NaC_3_H_5_O_2_; 0.157 g/liter). The pH of the synthetic produced water medium ranged from pH 6.6 to 6.7. Sterile carbon steel coupons in PEEK coupon holders were added as described above, and bottles were inoculated with 0.5% (vol/vol) of the original produced water and then incubated on rotary shakers (75 rpm) at 32°C for 11 weeks (Table S2).

### Kettle corrosion testing.

Customized corrosion kettles were used to simulate the influence of pipeline flow on CO_2_ corrosion and MIC (Fig. 3A). These reactors contained inward-facing corrosion coupons that were flush-mounted in an inert coupon holder cage (PEEK) and thus arranged around a central impeller with Rushton blades. The rotational speed was set to 500 rpm to correspond to an average coupon wall shear stress of 7 Pa (7 kg · m^−1^ · s^−2^), which was deemed representative of pipeline conditions based on multiphase flow modeling previously performed at this field. The glass reactor containing 1.5 liters of test brine was sealed air tight, with continuous purging of N_2_-CO_2_ (79:21; equivalent to 3 lb/in^2^ absolute CO_2_) through a diffusor (250 ml/min) and a gas outlet with refrigerated condenser tube to minimize loss of liquids via evaporation. Corrosion kettles were temperature-controlled at 32°C by means of a heating jacket with thermocouple. Control of pH was achieved through titration with 1 M HCl via syringe pumps coupled to online pH monitoring and a set point of pH 6.6. All tests contained four carbon steel coupons (5 cm^2^ each) and were operated for 7 weeks, unless otherwise stated.

Prior to test setup, corrosion kettles were rigorously cleaned and then “decontaminated” through liberal application of acetone. Sterile test conditions cannot be achieved in corrosion kettles. To minimize the growth of environmental contaminants, abiotic corrosion tests were amended with the antibiotics kanamycin (100 mg/liter), chloramphenicol (25 mg/liter), and tetracycline (20 mg/liter), which did not affect the acid gas corrosion rate or the coupon surface morphology in 7-day tests (see Fig. S5 in the supplemental material). Furthermore, vitamins, trace elements, phosphate, and ammonium were omitted from abiotic corrosion tests.

Corrosion testing was initially performed with the original PW-2016. This test included 1% (vol/vol) crude oil and was amended with sodium sulfate (Na_2_SO_4_; 10.4 mM) and spiked with an Fe^0^-containing, sulfate-amended PW pre-enrichment as additional inoculum (3% vol/vol). Testing under lithoautotrophic conditions was performed in synthetic produced water medium (no acetate, propionate, or oil), inoculated with the first passage of a lithoautotrophic enrichment culture (0.5% vol/vol) obtained from PW-2016. Finally, kettle corrosion testing was also performed with synthetic produced water medium containing sodium acetate and sodium propionate as organic electron donors for microbial metabolism and growth. This test was inoculated with the third subculture of an organotrophic, Fe^0^-containing enrichment (0.5% vol/vol) obtained from PW-2016 (Table S2).

### Gas chromatography.

For methane analysis, 10 ml headspace was sampled and quantified using an Agilent 490 Micro gas chromatograph with thermal conductivity detector and 10-μl injection loop. Chromatography was performed on a heated Agilent PoraPlot U column (50°C, 22.1 lb/in^2^, and 10 m), using argon as the carrier gas. Injector and sample line temperature were set to 100°C.

### Weight loss corrosion analysis.

API 5L X52 carbon steel coupons (≤0.26% C, ≤0.45% Si, ≤1.6% Mn, <0.03% P, and ≤0.03% S; balance Fe) were successively sonicated in hexane, acetone, and methanol (5 min each), dried in a stream of air, and placed under vacuum overnight. The weight of each coupon was measured three times on an analytical balance before placement into PEEK coupon holders. Coupon holders containing corrosion coupons were sterilized by soaking in ethanol (96%) for 10 min. Excess ethanol was dried with a stream of filtered N_2_ gas.

At the end of the test, corrosion products and other deposits were dissolved from coupons in a warm (70°C) 1.8 M HCl solution containing 2% propargyl alcohol (1 min). Wet coupons were then neutralized in saturated calcium hydroxide solution (30 s) and scrubbed with a nylon brush while being rinsed with deionized water. This procedure was performed twice. Coupons were then rinsed in acetone, dried under a stream of air, and placed under vacuum overnight. To determine weight loss, each coupon was weighed three times. Corrosion rates were calculated by considering the weight loss, exposed surface area, steel density (7.87 g/cm^3^), and exposure time.

### Localized corrosion analysis.

To visualize and measure localized corrosion, the topography of cleaned carbon steel coupons was mapped with a Keyence VR-3000K wide-area 3D profile measuring macroscope (Keyence Corp., Itasca, IL). Corrosion features (≥25 μm in depth) were quantified by referencing the original (precorrosion) surface height. Pitting corrosion rates, expressed as mm per year, were calculated by linear extrapolation.

### DNA sampling and extraction.

Coupon holders were removed from bioreactors (bottles or corrosion kettles) and thoroughly rinsed with sterile phosphate-buffered saline (PBS; 10 mM phosphate buffer, 2.7 mM KCl, and 137 mM NaCl [pH 7.4]) solution to remove loose (planktonic) microorganisms. Steel-attached biofilms were then sampled using sterile swabs (minimum of two swabs per coupon) and frozen at −80°C.

Frozen samples (pig debris or biofilm swabs) were shipped on ice to Microbial Insights, Inc. (Knoxville, TN), and DNA was extracted with the DNA Power soil total DNA isolation kits (Mo Bio Laboratories, Inc., Solana Beach, CA) according to the manufacturer’s instructions. DNA quantity and purity were assessed with a NanoDrop ND-1000 spectrometer (Thermo Fisher Scientific, Waltham, MA).

### Quantitative PCR.

Quantitative PCR was performed by Microbial Insights, Inc., on a QuantStudio 12K Flex real-time PCR system using a TaqMan universal PCR mastermix (Applied Biosystems, Grand Island, NY). For the quantification of bacteria and archaea, proprietary primers and TaqMan probes that target highly conserved regions of the 16S rRNA gene were used as described elsewhere (55, 56). Forward and reverse primers were obtained from Integrated DNA Technologies (Coralville, IA), while TaqMan probes were synthesized by Thermo Fisher Scientific.

For the qPCR assay targeting the large subunit of the specific [NiFe] hydrogenase (here referred to as *micH*) found in highly corrosive methanogenic archaea (see reference 24 and this study), a degenerated primer pair and TaqMan probe were designed based on conserved regions in homologous genes (Table 1 and Fig. S6 in the supplemental materials). Appropriate primer pairs and probes were selected using the Primer Express software (Thermo Fisher Scientific), and initial specificity was assessed by BLASTN searches against the NCBI nucleotide database (57). The qPCR was performed in a 20-μl volume using the TaqMan universal PCR mastermix. Forward and reverse primers, as well as the probe, were adjusted to a final concentration of 300 nM. For calibration and as a positive control, a synthetic fragment of the *micH* gene was used (see Fig. S6 and S7 in the supplemental material). The qPCR conditions were as follows: one cycle of 2 min at 50°C, followed by one cycle of 10 min at 95°C, 40 cycles of 15 s at 95°C, and 1 min at 60°C. The specificity of the qPCR assay was verified by Sanger sequencing of a final PCR product that confirmed the expected product sequence marked in Fig. S7. In addition, potential cross-reactions were excluded by running the newly developed qPCR assay on genome-sequenced cultures (see Table S3 in the supplemental material) that lack the special [NiFe] hydrogenase (*micH*).

### 16S rRNA gene amplicon sequencing and analysis.

16S rRNA gene amplicon sequencing was performed by Genewiz, Inc. (South Plainfield, NJ). DNA quality was assessed by agarose gel electrophoresis and quantified on a Qubit 2.0 fluorometer (Invitrogen, Carlsbad, CA). Barcoded amplicons covering the V3 to V5 region of the 16S rRNA gene were generated using Genewiz’s proprietary 16S MetaVx amplification protocols and library preparation kit. Sequencing libraries were evaluated on a 2100 Bioanalyzer (Agilent Technologies, Palo Alto, CA) and quantified on a Qubit fluorometer, as well as through qPCR. Paired-end sequencing (2 × 250 bp) of multiplexed DNA libraries was performed on a MiSeq platform (Illumina, San Diego, CA). In total, 18,109,298 paired-end reads were obtained.

The 16S rRNA data were analyzed using the QIIME pipeline (58). Joined reads were quality filtered to remove reads with a mean quality score of ≤20 and/or a read length of <200 bp and reads containing any ambiguous bases. Chimeric sequences were detected using the UCHIME algorithm (https://drive5.com/uchime/uchime_download.html) and the Ribosomal Database Program (RDP) Gold Database (59). After removal of chimeric sequences, the remaining sequences, which ranged from 300,398 to 1,816,578 reads, were assigned into operational taxonomic units (OTUs) with the VSEARCH (v1.9.6) clustering program and the SILVA 119 database (60), preclustered at 97% identity. Taxonomic categories were assigned to all OTUs at a confidence threshold of 0.8 by the RDP classifier. Between 65.1 and 99.3% of the reads were classified at the family level (for data shown in Fig. 1), and 89.1 and 99.2% were classified at the genus level (for data shown in Fig. 2). The raw reads have been deposited in the NCBI Sequence Read Archive (SRA) and can be accessed through BioProject accession number PRJNA644413.

### Shotgun metagenome sequencing and analysis.

Shotgun sequencing of extracted DNA was performed by Genewiz, Inc. The quality of extracted DNA was assessed by agarose gel electrophoresis and quantified on a Qubit 2.0 fluorometer. DNA libraries were prepared using the NEBNext Ultra DNA library prep kit (New England BioLabs, Inc., Ipswich, MA) according to the manufacturer’s instructions. The quality of DNA libraries was evaluated using an Agilent TapeStation and quantified using a Qubit fluorometer. Libraries were quantified by qPCR, loaded on to an Illumina HiSeq 4000 platform according to the manufacturer’s recommendations, and then sequenced using the paired-end (2 × 150 bp) configuration.

Subsequent bioinformatics analysis of paired-end sequencing reads was done in the KBase environment (61). Raw reads were quality trimmed and sequencing adapters removed by Trimmomatic (v0.36) using the sliding window option (window size of 4 and minimum quality score of 15), and reads shorter than 36 nucleotides were discarded from further analysis (62). The remaining 126 million paired-end reads were assembled with MetaSPAdes (v3.13.0) with default k-mer setting, read error correction enabled, and a final minimum contig size of 500 bp (63). The assembled contigs were binned using the MetaBAT2 (v1.7) binning algorithm with a minimum input contig length of 1,500 bp (64). The quality of the resulting 10 bins was evaluated using the CheckM (v1.0.18) software package (65). Taxonomy was assigned to individual bins using the Genome Taxonomy Database Toolkit (GTDB-Tk; v1.1.0), which utilizes domain-specific, concatenated marker protein reference trees, relative evolutionary divergence, and average nucleotide identity for taxonomic classification (66). Structural and functional annotation of binned contigs was achieved with NCBI’s prokaryotic annotation pipeline (67, 68). Additional annotations of unbinned contigs were obtained using the Prokka software package (69). Annotated MAGs and annotated unbinned contigs were screened for the presence or absence of certain genes by protein sequence searches via BLASTP (70). Hits were initially classified as positive by using an amino acid sequence identity threshold of ≥ 40%, an *E* value of ≥10^−5^, and an alignment length of ≥60%. The alignment of truncated genes at the ends of contigs that met the former thresholds were manually inspected.

### Data availability.

The reads used for assembly and binning have been deposited to the NCBI SRA, and the annotated draft genomes have been deposited at GenBank under BioProject accession number PRJNA644413.

## Supplementary Material

Supplemental file 1
