# Supplementary material for: Severe Corrosion of Carbon Steel in Oil Field Produced Water Can Be Linked to Methanogenic Archaea Containing a Special Type of [NiFe] Hydrogenase
Source: Appl Environ Microbiol. 2021 Jan 15;87(3):e01819-20. doi: 10.1128/AEM.01819-20 (PMC7848899; doi:10.1128/AEM.01819-20)
Supplement: Supplemental file 1 [file AEM.01819-20-s0001.pdf]

Table S1 Top 25 hits for a protein sequence BLAST (BlastP) search of MicH against NCBI's non-redundant protein database.

Putative function	Closest taxonomic classification	Total BlastP score	Query coverage (%)	E-value	Sequence identity (%)	Accession No.
nickel-dependent hydrogenase large subunit	*Methanococcus maripaludis* OS7	1026	100	0.0E+00	100.0	WP_119846095.1
hypothetical protein	*Methanobacterium* sp.	946	98	0.0E+00	93.5	RJS49388.1
hypothetical protein	*Methanobacteriales*	887	98	0.0E+00	90.7	PKL67834.1
nickel-dependent hydrogenase large subunit	*Methanobacterium congolense Buetzberg*	884	98	0.0E+00	90.7	WP_071906935.1
hypothetical protein	*Methanopyrus* sp. SNP6	340	96	9.0E-108	40.7	WP_148689234.1
hypothetical protein	*Bacteria*	176	96	2.0E-45	28.5	NLI99272.1
Ni/Fe hydrogenase subunit alpha	Ca. *Korarchaeota*	170	96	2.0E-43	28.8	HDD68771.1
Fe hydrogenase	*Phycisphaerae*	170	98	4.0E-43	29.0	KPK74792.1
Ni/Fe hydrogenase subunit alpha	*Desulfosporosinus* sp. Sb-LF	168	96	2.0E-42	28.0	WP_135380469.1
Ni/Fe hydrogenase subunit alpha	*Nitrosomonadales*	168	96	2.0E-42	29.5	PWB56486.1
Ni/Fe hydrogenase subunit alpha	*Sulfuritalea hydrogenivorans*	167	98	2.0E-42	29.4	WP_041099025.1
Ni/Fe hydrogenase subunit alpha	*Archaea*	167	96	4.0E-42	28.6	HEA10786.1
Ni/Fe hydrogenase subunit alpha	*Nitrosospira* sp. Nsp13	166	96	7.0E-42	29.2	WP_090907184.1
Ni/Fe hydrogenase subunit alpha	*Nitrosospira multiformis*	166	96	8.0E-42	28.7	WP_107761584.1
Ni/Fe hydrogenase subunit alpha	candidate division WOR-3	165	97	1.0E-41	28.2	HHR48257.1
Ni/Fe hydrogenase subunit alpha	candidate division WOR-3	165	97	2.0E-41	27.9	HGU47450.1
NADP oxidoreductase	*Nitrosomonadales*	165	96	2.0E-41	29.0	ODT84812.1
Ni/Fe hydrogenase subunit alpha	*Nitrosomonadales*	165	96	2.0E-41	29.5	TFH12556.1
Ni/Fe hydrogenase subunit alpha	candidate division WOR-3	164	97	4.0E-41	28.7	HGK62977.1
Ni/Fe hydrogenase subunit alpha	Ca. *Bathyarchaeota*	163	96	6.0E-41	28.5	HGD66108.1
Ni/Fe hydrogenase subunit alpha	*Nitrosospira lacus*	164	96	8.0E-41	29.9	WP_004174406.1
nickel-dependent hydrogenase large subunit	*Aquisphaera* sp. JC650	163	98	9.0E-41	29.4	WP_152053939.1
F420-non-reducing hydrogenase subunit A	*Lokiarchaeum* sp. GC14_75	163	96	9.0E-41	27.9	KKK40681.1
Ni/Fe hydrogenase subunit alpha	*Firmicutes*	163	98	9.0E-41	27.9	PKM81450.1
Ni/Fe hydrogenase subunit alpha	*Anaerolineae*	163	96	1.0E-40	30.3	HGY48290.1

Table S2 Additional details for corrosion tests shown in the main manuscript. For easier identification the description matches the labelling of tests in individual figures.

Experiment	Test No.	Description	Medium	Inoculum	Incubation temperature (℃)	Test duration	Weight loss corrosion rate (mm/yr)	Shown in manuscript
Tempeature dependency in sulfate-free produced water	1	Sterile at 32℃	Nigerian produced water collected in 2014	Sterile	32	13 weeks	0.00 ± 0.00	Figure 2A - C
	2	Sterile at 60℃	Nigerian produced water collected in 2014	Sterile	60	13 weeks	0.02 ± 0.00	Figure 2A - C
	3	32℃	Nigerian produced water collected in 2014	Microorganism contained in produced water	32	13 weeks	0.19 ± 0.09	Figure 2A - C
	4	32℃	Nigerian produced water collected in 2014	Microorganism contained in produced water	32	13 weeks	0.19 ± 0.08	Figure 2A - C
	5	60℃	Nigerian produced water collected in 2014	Microorganism contained in produced water	60	13 weeks	0.02 ± 0.01	Figure 2A - C
	6	60℃	Nigerian produced water collected in 2014	Microorganism contained in produced water	60	13 weeks	0.03 ± 0.01	Figure 2A - C
Sulfate amendment in original and synthetic produced water	7	Sterile medium	Synthetic produced water	Sterile	32	6 weeks	0.03 ± 0.00	Figure 2D - F
	8	Sterile medium	Synthetic produced water	Sterile	32	6 weeks	0.03 ± 0.01	Figure 2D - F
	9	Produced water	Nigerian produced water collected in 2016	Microorganism contained in produced water	32	11 weeks	0.31 ± 0.04	Figure 2D - F
	10	Produced water	Nigerian produced water collected in 2016	Microorganism contained in produced water	32	11 weeks	0.15 ± 0.04	Figure 2D - F
	11	Organotrophic medium	Synthetic produced water	0.5% (v/v) of 2016 produced water	32	11 weeks	0.06 ± 0.02	Figure 2D - F
	12	Organotrophic medium	Synthetic produced water	0.5% (v/v) of 2016 produced water	32	11 weeks	0.07 ± 0.02	Figure 2D - F
	13	Lithotrophic medium	Synthetic produced water	0.5% (v/v) of 2016 produced water	32	11 weeks	0.08 ± 0.04	Figure 2D - F
	14	Lithotrophic medium	Synthetic produced water	0.5% (v/v) of 2016 produced water	32	11 weeks	0.76 ± 0.15	Figure 2D - F
Corrosion kettle tests	15	Sterile medium	Synthetic produced water	Sterile	32	7 weeks	0.34 ± 0.01	Figure 3B
	16	Produced water	Nigerian produced water collected in 2016	Microorganism contained in produced water	32	7 weeks	0.54 ± 0.14	Figure 3B
	17	Lithotrophic medium	Synthetic produced water	0.5% (v/v) from lithotrophic test No. 14	32	7 weeks	1.62 ± 0.72	Figure 3B
	18	Organotrophic medium	Synthetic produced water	0.5% (v/v) organotrophic enrichment (3rd transfer) from 2016 collected produced water (not shown here)	32	7 weeks	0.07 ± 0.00	Figure 3B

Table S3 Microorganisms used as negative controls to evaluate *micH* qPCR assay.

Organism name	Genome Accession No.	Origin	Country	Genome status	*micH* detected
Archaeoglobus fulgidus ATCC 49558	NC_000917.1	Hot Spring	Italy	Complete	−
Burkholderia thailandensis ATCC 700388	NC_007651.1	Rice field soil	Thailand	Complete	−
Desulfovibrio vulgaris ATCC 29579	NC_002937.3	Wealden clay	United Kingdom	Complete	−
Eubacterium limosum ATCC 8486	NZ_CP019962.1	Human intestinal content	Germany	Complete	−
Geobacter metallireducens ATCC 53774	NC_007517.1	Fresh water sediment, Maryland	United States	Complete	−
Geobacter sulfurreducens ATCC 51573	NC_002939.5	Surface sediment, Oklahoma	United States	Complete	−
Methanobacterium bryantii ATCC 33272	NZ_LMVM00000000.1	Syntrophic culture of "*Methanobacterium omelianskii*"	NA	Draft	−
Methanocaldococcus jannaschii ATCC 43067	NC_000909.1	Submarine hydrothermal vent	Pacific Ocean, East Pacific Rise	Complete	−
Methanococcus voltae ATCC BAA-1334	CP002057.1	Salt marsh, Florida	United States	Complete	−
Methanoculleus bourgensis ATCC 43281	NC_018227.2	Tannery sewage sludge	France	Complete	−
Methanomicrobium mobile ATCC 35094	NZ_JOMF00000000.1	Bovine rumen	United States	Draft	−
Nitrosomonas europaea ATCC 19718	NC_004757.1	NA	NA	Complete	−
Roseobacter denitrificans ATCC 33942	NC_008209.1	Seaweed	NA	Complete	−
Shewanella denitrificans ATCC BAA-1090	NC_007954.1	Water column, 130 m depth	Baltic Sea	Complete	−
Thiobacillus denitrificans ATCC 25259	NC_007404.1	Soil, Texas	United States	Complete	−

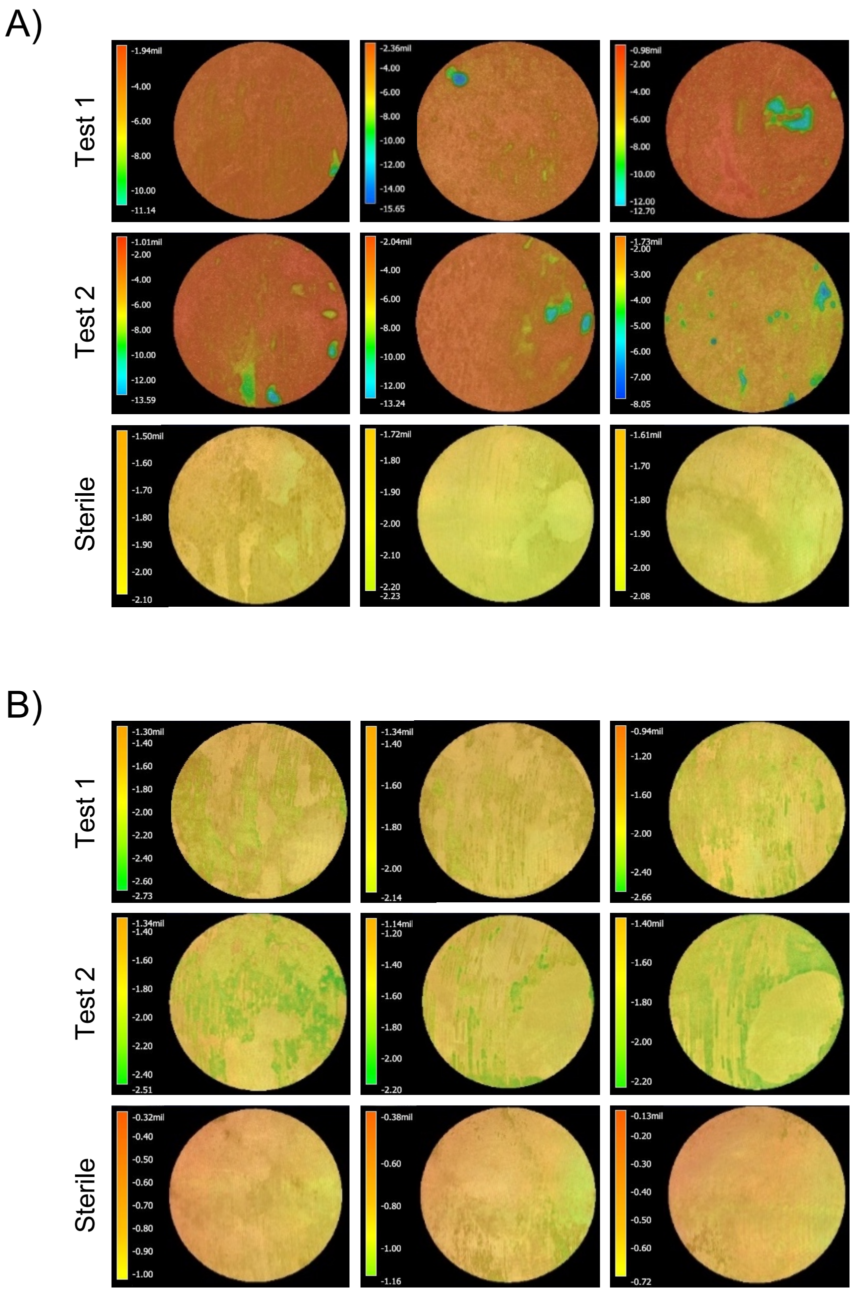

Figure S1 Surface topography of cleaned X52 carbon steel weight loss corrosion coupons after exposure to produced water at A) 32°C and B) 60°C. See Figure 2A for corresponding weight loss corrosion data.

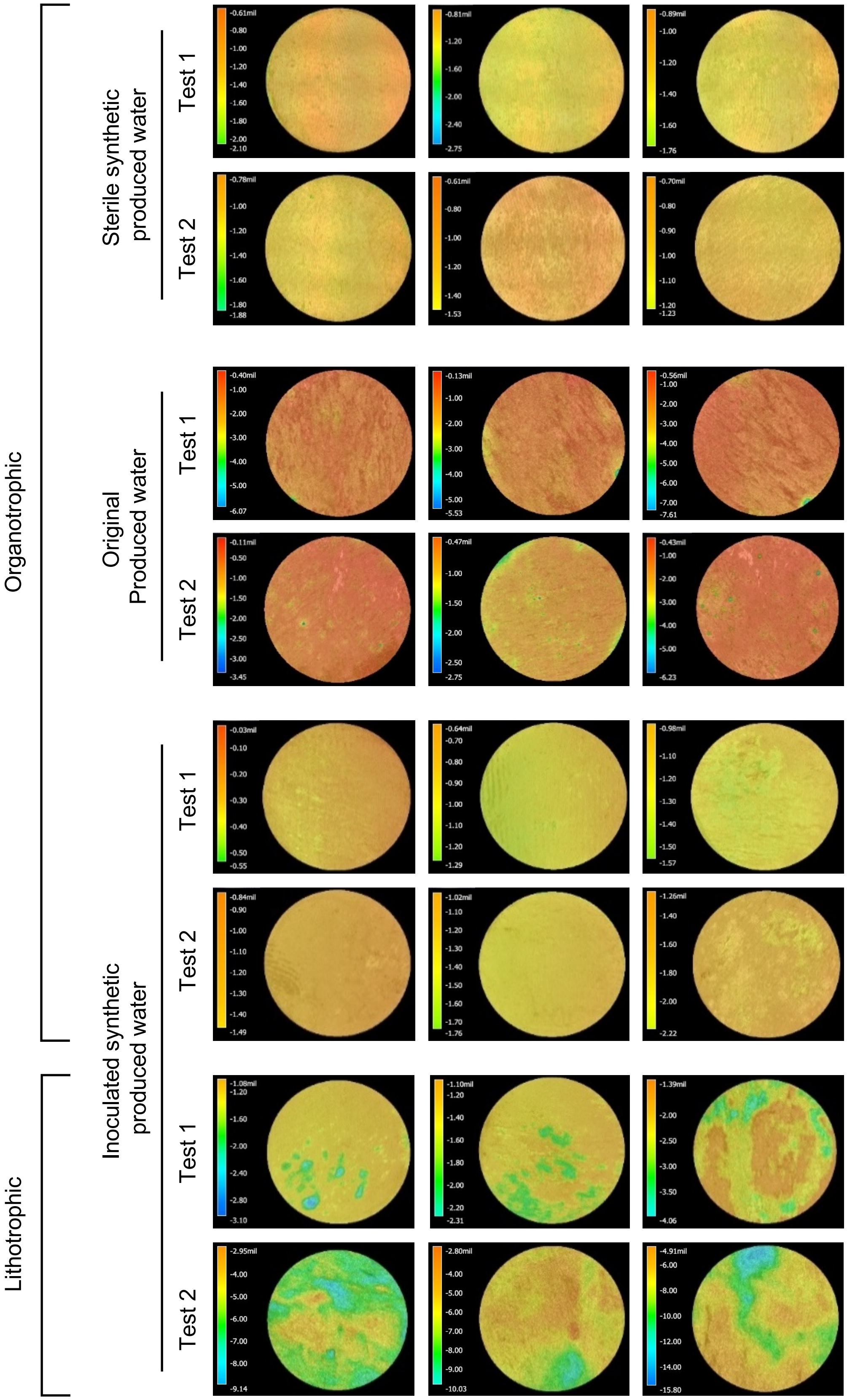

Figure S2 Surface topography of cleaned X52 carbon steel weight loss corrosion coupons after exposure to original produced water, sterile or inoculated synthetic produced water at 32°C under organotrophic or lithotrophic conditions. See Figure 2D for corresponding weight loss corrosion data.

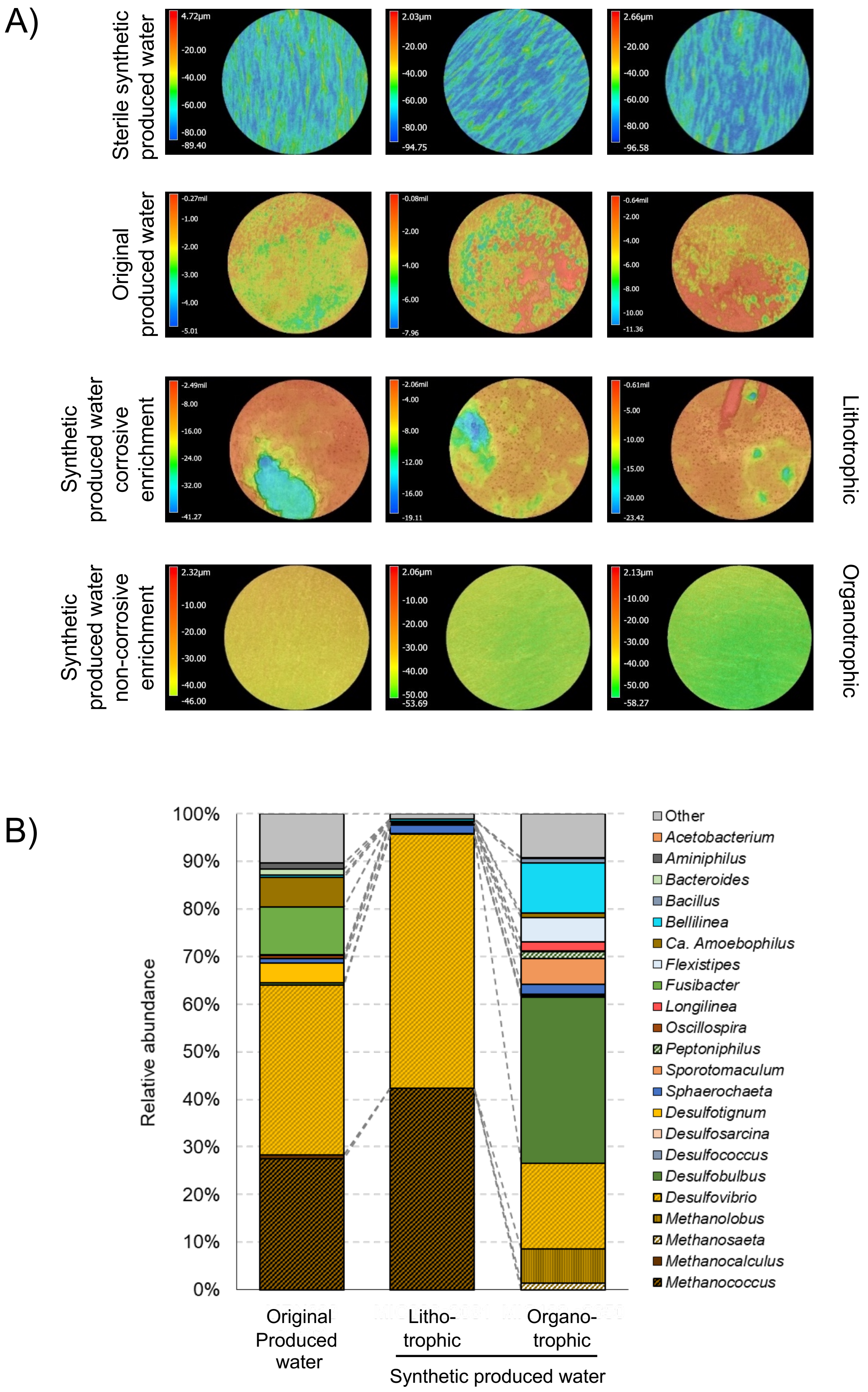

Figure S3 A) Surface topography of cleaned X52 carbon steel weight loss corrosion coupons after exposure to produced water or synthetic produced water under simulated pipeline conditions in customized reactors. See Figure 3D for corresponding weight loss corrosion data. B) Microbial community composition on carbon steel coupon surfaces after incubation in customized reactors under simulated pipeline conditions. See Figure 3D for weight loss corrosion rates.

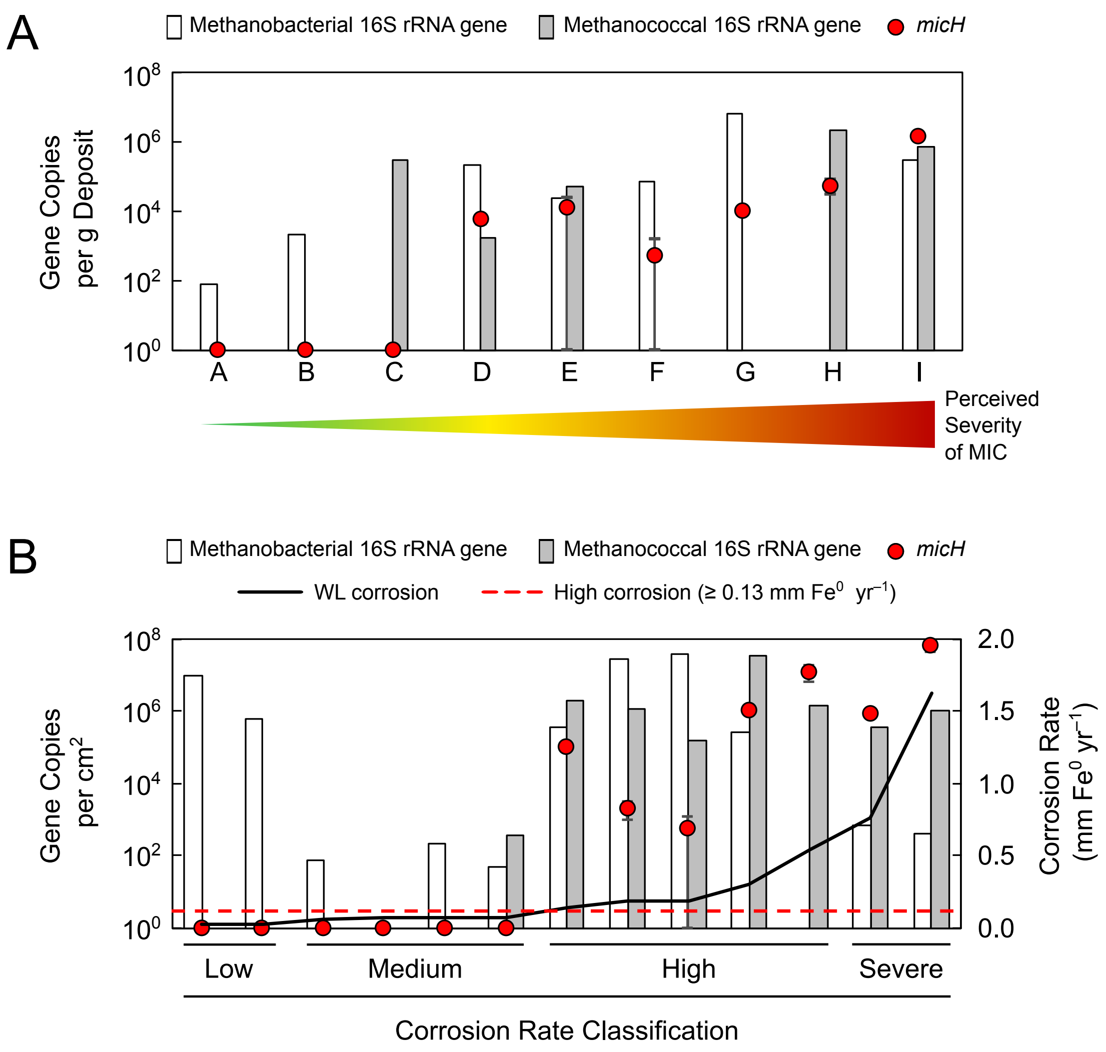

Figure S4 Comparison of corrosion rate, micH gene copies and estimated methanococcal and methanobacterial 16S rRNA gene copies in A) pigging debris samples shown in Figure 1 and B) laboratory tests shown in Figures 2 and 3. Genus specific estimated 16S rRNA gene copies were calculated from total 16S rRNA gene copy numbers obtained by qPCR and relative community composition data from 16S rRNA gene amplicon sequencing. The error bars associated with *micH* qPCR results depict the standard deviation of technical replication (n=3).

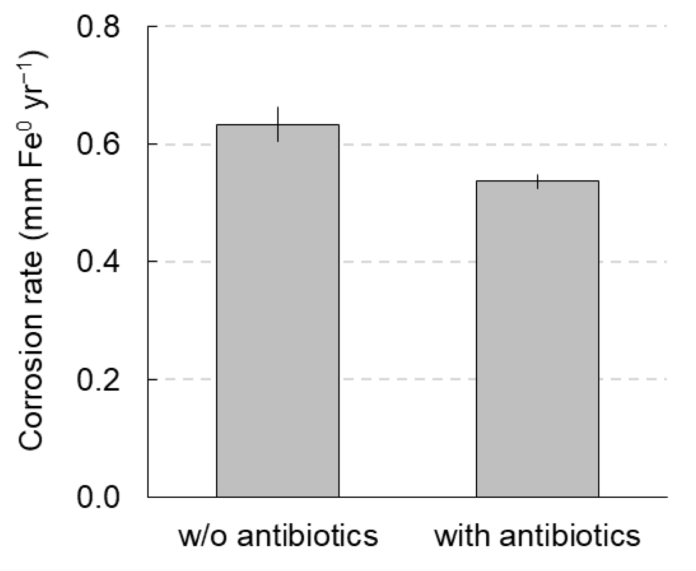

Figure S5 Comparison of corrosion rate in kettle tests using sterile synthetic produced water treated with or without (w/o) antibiotics.

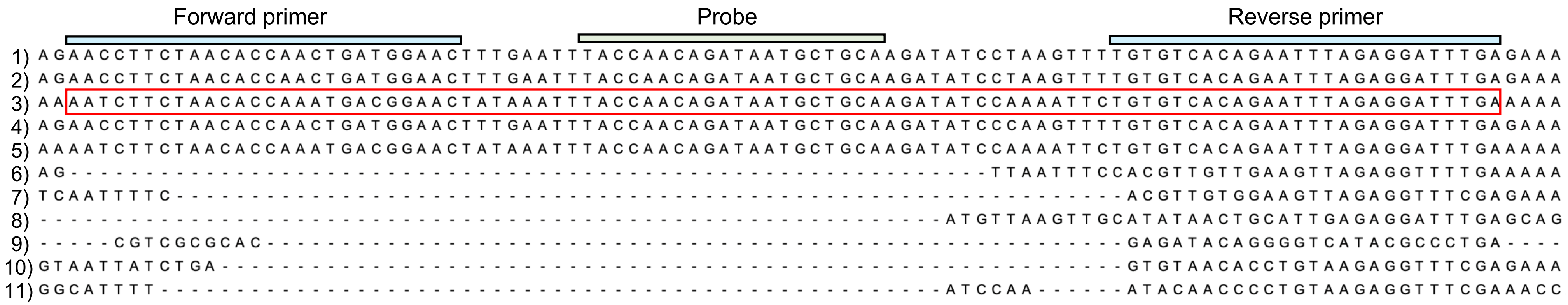

Figure S6 Alignment of *micH* gene region used for primer and probe design (sequences 1 – 5). Primer and probe binding sites are shaded with different colors and the synthetic fragment used for quantification is edged in red. Alignment of large subunits of remotely related hydrogenases found in methanogens not carrying the *micH* gene (sequence 6 – 11) showed the desired specificity of the selected primers and probe. Sequence 1) *micH* (H0S71_01960) *Methanococcus maripaludis* MIC098Bin5 metagenome (JACCQJ000000000); 2) *micH* (MMOS7_11600) *Methanococcus maripaludis* OS7 (AP011528.1); 3) *micH* (CVV28_03670) *Methanobacteriales* groundwater metagenome (PGYO01000002.1); 4) *micH* (CIT03_03910) *Methanobacterium* bioreactor metagenome (NPZC01000005.1); 5) *micH* (MCBB_1253) *Methanobacterium congolense* Buetzberg (LT607756.1); 6) MMP_RS04285 *Methanococcus maripaludis* S2 (NC_005791.1); 7) MVOL_RS02900 *Methanococcus voltae* A3 (NC_014222.1); 8) MJ_RS06365 *Methanocaldococcus jannaschii* DSM 2661 (NC_000909.1; 9) MBBA_RS09640 *Methanoculleus bourgensis* (NZ_LT549891.1); 10) MVOL_RS02915 *Methanococcus voltae* A3 (NC_014222.1); 11) MMP_RS07120 *Methanococcus maripaludis* S2 (NC_005791.1); 12) Partial sequence of a *micH* qPCR product from a corrosive biofilm sample retrieved by Sanger sequencing. Sequences were aligned with MUSCLE in MEGA7 (Reference: Kumar S, Stecher G, Tamura K (2016) MEGA7: Molecular Evolutionary Genetics Analysis Version 7.0 for BIGGER Datasets. Mol Biol Evol 33: 1870–1874. doi:10.1093/molbev/msw054).

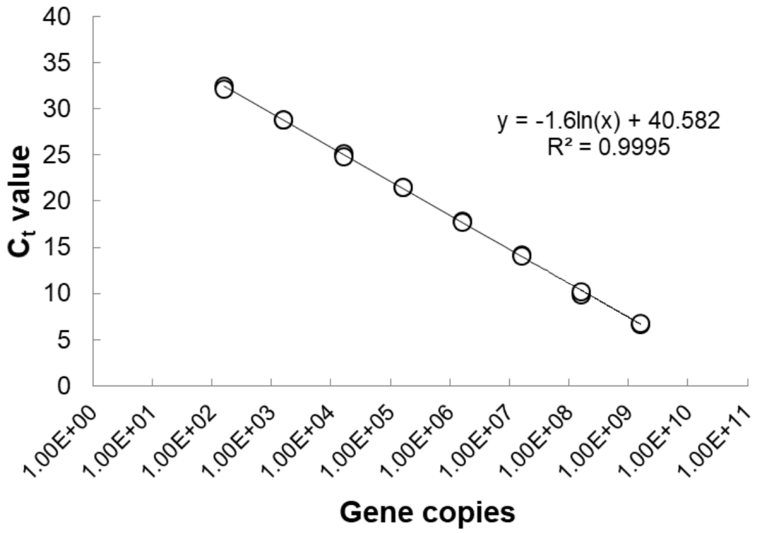

Figure S7 Calibration curve of quantitative PCR using a synthetic fragments of *micH* gene as target and the newly developed primer pair and Taqman™ probe.
